# Diagnostic single nucleotide polymorphism markers to identify hybridization between dromedary and Bactrian camels

**DOI:** 10.1007/s12686-015-0420-z

**Published:** 2015-01-13

**Authors:** Emily Ruiz, Elmira Mohandesan, Robert R. Fitak, Pamela A. Burger

**Affiliations:** Institut für Populationsgenetik, Vetmeduni Vienna, Veterinärplatz 1, 1210 Vienna, Austria

**Keywords:** Single nucleotide polymorphisms (SNPs), Hybrid camel, Next generation sequencing (NGS), *Camelus bactrianus*, *Camelus dromedarius*, Introgression

## Abstract

The technique to produce hybrid *Tulu* or *Nar* camels from crosses between dromedary and Bactrian camels is common throughout Middle Eastern and Central Asian countries. Formerly, these hybrids were highly valued as strong and persistent pack animals but today are bred to improve milk or wool quality in the respective species and for camel wrestling. We developed a diagnostic single nucleotide polymorphism (SNP) panel to identify cryptic ancestry in F_1_ hybrids and their backcrosses by selecting loci from whole genome data, which were fixed for different alleles in either dromedary or domestic and wild Bactrian camel. With this SNP panel we are able to identify the hybridization patterns in camels with uncertain origins, support hybrid breeding management and to detect potential rare dromedary introgression in the last wild Bactrian camels in Mongolia and China.

**Electronic supplementary material:**

The online version of this article (doi:10.1007/s12686-015-0420-z) contains supplementary material, which is available to authorized users.

## Technical note

Anthropogenic hybridization between livestock populations or between domestic species and their wild relatives has been practiced since the early phases of domestication. In Old World camelids, hybridization between Bactrian camels (*Camelus bactrianus*) and dromedaries (*Camelus dromedarius*) was associated with the transportation of goods along the multiple routes of the Silk Road. This practice intended to produce animals with the robustness of the Bactrian camel, the endurance of dromedary, and the ability to tolerate sharply contrasting climatic conditions (Wilson 1984). Today, hybridization facilitates improved milk and wool yield in hybrid *Tulu* or *Nar* camels from Middle Eastern and Central Asian countries. Commonly, two hybridizing methods are recognized, *Kurt*-*nar* (dromedary female × Bactrian male) and *Kez*-*nar* (Bactrian female × dromedary male) followed by F_1_ backcrossing with either dromedary for increased milk productivity or Bactrian camel for wool and cold resistance (Faye and Konuspayeva 2012). F_2_ hybrids (F_1_ × F_1_) in Old World camels are usually not favoured because of a difficult character and weak progeny performance (Faye and Konuspayeva 2012). In western regions of Turkey, a relished sport is camel wrestling, where prized male *Tulus* compete against each other in heavily regulated fights (Vedat 2010). In this study, we developed diagnostic single nucleotide polymorphisms (SNPs) to identify F_1_ hybrids between dromedary and Bactrian camels and their backcrosses. These markers will facilitate the validation of non-invasive or historic samples with unclear or otherwise cryptic ancestry.

As part of an on-going research project, we sequenced the genomes of 25 Old World camelids [*C. dromedarius* (n = 9), *C. ferus* (n = 9), *C. bactrianus* (n = 7)] and aligned them to the *C. ferus* CB1 genome assembly (Genbank ID: GCA_000311805.2). To verify their species identity, these source individuals had previously been genotyped at 18 nuclear microsatellite loci and Sanger sequenced for 800 bp of mitochondrial DNA (unpublished data). We identified SNPs according to the guidelines presented by Van der Auwera et al. (2013) and selected polymorphisms that were fixed for different alleles between *C. dromedarius* and both Bactrian camel species, *C. bactrianus* and *C. ferus* (see ‘Supplementary Methods’ in Online Resource 1). We designed PCR primers around each SNP using PRIMER3 (Untergasser et al. 2012) and selected 26 primer pairs (Online Resource 2) for validation.

We tested the 26 candidate loci using PCR and Sanger sequencing (see ‘Supplementary Methods’ in Online Resource 1) on a panel of control individuals including three Bactrian camels, three dromedaries, two hybrids (F_1_ and F_1_ backcross) and a two-humped individual with uncertain genetic background. All 26 candidate loci produced PCR products of the expected sizes, but we selected for sequencing only those 12 loci which produced single strong bands over consistent conditions (Table 1). Upon sequencing, all 12 loci were diagnostic between dromedaries and Bactrian camels (Table 2). However, in the F_1_ hybrid (Hyb56) only 10 (83.3 %) of the diagnostic loci were heterozygous and the remaining two loci were homozygous for the Bactrian allele (Table 2). This suggests that at these two loci (HP264 and HP288) the Bactrian allele may be present at low frequency in dromedaries as a result of being a shared ancestral polymorphism (not diagnostic) or from historical hybridization between the two species. The F_1_ backcross (Hyb55) was heterozygous for two (16.7 %) of the diagnostic loci and homozygous for the Bactrian allele in the remaining loci. In the two-humped individual with uncertain origin (DC575), four (33.3 %) of the diagnostic loci were heterozygous, demonstrating hybrid ancestry in this animal (Table 2).

**Table 1:** Primer sequences and information for the 12 diagnostic loci

Locus	Primer sequence (5′–3′)^a^	Scaffold^b^	Position^b^	Alleles	T^m^	Length
HP206	TGTCAGACTGTTAGGCATTGC CATCCAAGTCTCCATCTAACCC	NW_006210212.1	501206	C/G	57	125
HP379	AGGATGCCATCATGTCAGG GAGGGAGCTCTCATGAATAGG	NW_006210464.1	1151379	G/A	58	150
HP405	CCAGGAGCTTTTCGAGTAGC CAGCACAGAGAACTCACTGC	NW_006211106.1	5030405	G/C	59	125
HP429	GCAGGCATACAAACTAACCC GCTTTTCTTTCTGGCTCAGG	NW_006210666.1	2288429	A/C	57	125
HP458	TGTGACCAGACAGACCAAGG TGTGGCTTAGGGTCTTTATGG	NW_006210457.1	11458	T/C	58	140
HP501	GAATAGATTGGGGAGCAAGC CTCTTCTCCATCCCTATGGC	NW_006211169.1	218501	A/T	59	125
HP597	ATGAACAGTTTGGGTTTGGG CGCGATGTCACCTTTATAGG	NW_006211126.1	6065597	A/C	59	125
HP633	GCATGTAGAAGGTTTGCATAGG CAGCCTTTCTTGCATCTGG	NW_006210489.1	4279633	G/C	57	125
HP900	CCACATGCTCAGGTATCTGG GGGATTCCTTGTGCTACAGC	NW_006211075.1	294900	G/C	59	125
HP930	CTCCCAGGAAACAAAAGTCC TTTGGGAGTGTTCTGTCTGC	NW_006210745.1	3459930	C/A	59	125
HP264	TGGACAGAAACTTTGTGTCTCC TTTGGTAAGGGCATGAATCC	NW_006211022.1	519264	T/C	59	125
HP288	GTCTATGAGGGCGTTTCTGC CAGCCTTCTTGTTCTGTTCG	NW_006211252.1	214288	T/A	59	125

See Online Resource 2 for additional details on these and the remaining 14 primers examined

T_m_: primer annealing temperature

Length: PCR product length

^a^Left (forward) primer given above the right (reverse) primer

^b^Accession number from GenBank and position of the SNP in the scaffold

**Table 2:** Sanger genotypes for the 12 loci sequenced

Sample ID	Species	Location	HP206 (G|C)	HP379 (A|G)	HP405 (C|G)	HP429 (C|A)	HP458 (C|T)	HP501 (T|A)	HP597 (C|A)	HP633 (C|G)	HP900 (C|G)	HP930 (A|C)	HP264^a^ (C|T)	HP288^a^ (A|T)
Drom155	*C. dromedarius*	Australia	GG	AA	CC	CC	CC	TT	CC	CC	CC	AA	CC	AA
Drom214	*C. dromedarius*	Syria	GG	AA	CC	CC	CC	TT	CC	CC	CC	AA	CC	AA
Drom814	*C. dromedarius*	Sudan	GG	AA	CC	CC	CC	TT	CC	CC	CC	AA	CC	AA
DC69	*C. bactrianus*	Mongolia	CC	GG	GG	AA	TT	AA	AA	GG	GG	CC	TT	TT
DC158	*C. bactrianus*	Austria	CC	GG	GG	AA	TT	AA	AA	GG	GG	CC	TT	TT
DC352	*C. bactrianus*	Iran	CC	GG	GG	AA	TT	AA	AA	GG	GG	CC	TT	TT
Hyb56	F_1_ hybrid^b^	Kazakhstan	GC	GA	GC	AC	CT	TA	AC	GC	GC	AC	TT	TT
Hyb55	F_1_ backcross^b^	Kazakhstan	CC	GA	GG	AA	CT	AA	AA	GG	GG	CC	TT	TT
DC575	uncertain origin	Kazakhstan	CC	GA	GG	AC	TT	AA	AA	GC	GG	CC	CT	TT

The parentheses indicate the reference alleles (dromedary allele | Bactrian allele) identified from the whole-genome sequencing

Location: sample’s country of origin

HP206 ff.: SNP locus names

^a^These loci show patterns inconsistent with a diagnostic SNP in the F1 hybrid

^b^F1 (dromedary female × Bactrian male); F1 backcross (F1 female × Bactrian male)

With this panel of diagnostic SNPs it is possible to identify F_1_ hybrids between dromedary and Bactrian camels and to differentiate F_1_ backcrosses to either parental species. Thus, we can validate the status of individuals with unclear parental origins in regions where the two species are extensively crossbred (e.g. Kazakhstan, Turkey). Because we selected short PCR fragments (≤150 bp) the panel can be applied in non-invasive, historical and archaeological (ancient) samples, where the morphology of the preserved materials cannot retrieve unambiguous classification (Gallik et al. 2015). Moreover, with this marker set we will be able to detect potential, rare dromedary introgression in Bactrian camel populations in Mongolia and China. As hybridization between domestic Bactrian camels and their wild relatives has been demonstrated (Silbermayr and Burger 2012; Silbermayr 2009), the threat for dromedary introgression into the last wild two-humped camels (*C. ferus*) in the Mongolian Gobi (Yadamsuren et al. 2012) and Chinese Taklimakan and Lop Nor deserts (Lei et al. 2012) is small but existing. Therefore, this primer set is an important contribution to the conservation of the genetic integrity of this critically endangered species.

## Electronic supplementary material

Below is the link to the electronic supplementary material.

Supplementary material 1 (PDF 112 kb)

Supplementary material 2 (PDF 32 kb)

Supplementary material 3 (PDF 61 kb)
