# Supplementary material for: Diagnostic single nucleotide polymorphism markers to identify hybridization between dromedary and Bactrian camels
Source: Conserv Genet Resour. 2015 Jan 13;7(2):329–32. doi: 10.1007/s12686-015-0420-z (PMC4486411; doi:10.1007/s12686-015-0420-z)
Supplement: Supplementary file 1 — Supplementary material 1 (PDF 112 kb) [file 12686_2015_420_MOESM1_ESM.pdf]

Online Resource 1

Diagnostic single nucleotide polymorphism markers to identify hybridization between dromedary and Bactrian camels
In *Conservation Genetics Resources*
Emily Ruiz, Elmira Mohandesan, Robert R. Fitak and Pamela A. Burger*
Institut für Populationsgenetik, Vetmeduni Vienna, Veterinärplatz 1, 1210 Vienna, Austria
*Corresponding author
pamela.burger@vetmeduni.ac.at

Supplementary Methods

Whole-genome sequencing

The dataset consisted of whole-genome shotgun sequences from three species of Old World camels (*Camelus dromedarius* [n = 9], *Camelus ferus* [n = 9], and *Camelus bactrianus* [n = 7]) as part of an on-going study in our laboratory. For each individual we extracted DNA using Master Pure™ DNA purification kit for blood (Epicentre version III) and performed paired-end sequencing on a single lane of an Illumina HiSeq (Illumina, USA) with a mean insert size of 500 bp. We trimmed the 3' end of sequence reads to a minimum phred-scaled base quality score of 20 and minimum length of 50 bp using POPOOLATION v1.2.2 (Kofler et al. 2011). We aligned the trimmed reads to the *C. ferus* CB1 genome assembly (Genbank ID: GCA_000311805.2) using BWA v0.6.2 (Li and Durbin 2009). We removed all duplicate reads using PICARD v1.89 (http://picard.sourceforge.net) and filtered all alignments to contain only unambiguously mapped and properly paired reads using SAMTOOLS v0.1.19 (Li et al. 2009). We realigned reads near insertions/deletions and recalibrated the base quality scores according to the Genome Analysis Toolkit (GATK) Best Practices Pipeline (Van der Auwera et al. 2013).

SNP identification

We identified SNPs from the whole-genome alignments using the GATK 'HaplotypeCaller' employing a minimum phred-scaled SNP quality score of 30. We

excluded SNPs that matched the following GATK filter criteria: depth of coverage (DP) > 750 (~30X/individual), SNP quality by depth (QD) < 2.0, strand bias (FS) > 60.0, mapping quality (MQ) < 40.0, mapping quality rank sum test (MQRankSum) < -12.5, and read position bias (ReadPosRankSum) < -8.0. We excluded individual genotypes if the genotype quality score (GQ) was less than 10.0. We also excluded SNPs genotyped in less than 80% of individuals, within 200 bp of the end of a scaffold, and found in repeat-masked locations in the genome using BEDTOOLS v2.17.0 (Quinlan and Hall 2010). For our final SNP dataset we selected only those loci fixed between *C. dromedarius* and both *C. bactrianus* and *C. ferus*.

Primer design

For each SNP we selected 200 bp of flanking sequence in both directions (401 bp total) using the 'fill-fs' command in VCFTOOLS v0.1.12a (Danecek et al. 2011). We designed PCR primers for each SNP using PRIMER3 v2.3.6 (Untergasser et al. 2012) employing a variety of criteria to customize the primer selection (see Appendix SA). We targeted a fragment size between 80 and 200 bp that contained the SNP and at least 20 bp of flanking sequence. We randomly selected 26 primer pairs for testing that amplified a fragment ≤ 150 bp in length and were found on different scaffolds.

DNA extraction and PCR amplification

We obtained blood, liver and hair samples from presumably purebred *C. dromedarius* (n = 3) and *C. bactrianus* (n = 3), in addition to a known F_1 hybrid (dromedary female x Bactrian male), a F_1 backcross (F_1 female x Bactrian male), and a two-humped individual with unknown genetic background, during routine veterinary procedures (see main text Table 1). We extracted DNA from blood using the Master Pure™ DNA Purification Kit (Epicentre

version II). We used the manufacturer's recommendations with the following changes: i) in the first centrifugation we used one minute at maximum speed, ii) in the fourteenth step we increased the centrifugation time to 20 minutes, and iii) made a final elution in 100 uL TE instead of 35 uL. We quantified all DNA extractions with the Qubit™ fluorometer (Invitrogen™) and diluted samples to ~2.0 ng/μl.

The amplification reactions were carried out using 1.5 μl of DNA, 1X PCR buffer I (Agrobiogen, Hilgertshausen-Tandern, DE), 1 mg/ml of BSA, 2 mM $MgCl_2$, 0.25 mM each dNTPs, 0.6 μM of forward and reverse primer, and 1 unit of *Taq* DNA polymerase (Biotaq; Agrobiogen) in a final reaction volume of 25 μl. PCR amplification was performed using a 2720 thermal cycler AB (Applied Biosystems, Vienna, AT) with the following cycling conditions: an initial denaturation at 95 °C for 5 min, then 30 cycles of 95 °C for 30 sec, 57-59 °C for 45 sec (57 °C: HP206, HP429, HP633; 58 °C: HP458, HP379; 59 °C: HP405, HP900, HP288, HP597, HP264, HP930, HP501) and 72 °C for 45 sec. A final extension step at 95 °C for 5 min was included. We checked amplification success through electrophoresis in a 1.5% agarose gel stained with ethidium bromide (50 ng/ml) and visualized over ultraviolet light. PCR products were sequenced in both directions at LGC Genomics (www.lgcgroup.com, Middlesex, UK) using the same amplification primers. We visually inspected all chromatograms for polymorphisms using CodonCode Aligner v3.7.1.2. (CodonCode Corporation, Centerville, USA).

References

Danecek, P., Auton, A., Abecasis, G., Albers, C. A., Banks, E., DePristo, M. A., Handsaker, R. E., Lunter, G., Marth, G. T., Sherry, S. T., McVean, G., Durbin, R., and 1000 Genomes Project Analysis Group. 2011. The cariant call format and VCFtools. *Bioinformatics* 27, 2156-2158.

Kofler, R., Orozco-terWengel, P., De Maio, N., Pandey, R.V., Nolte, V., Futschik, A., Kosiol, C. and Schlotterer, C., 2011. PoPoolation: a toolbox for population genetic analysis of next generation sequencing data from pooled individuals. *PLoS One* 6, e15925.

Li, H. and Durbin, R., 2009. Fast and accurate short read alignment with Burrows-Wheeler transform. *Bioinformatics* 25, 1754-1760.

Li, H., Handsaker, B., Wysoker, A., Fennell, T., Ruan, J., Homer, N., Marth, G., Abecasis, G. and Durbin, R., 2009. The Sequence Alignment/Map format and SAMtools. *Bioinformatics* 25, 2078-2079.

Van der Auwera, G.A., Carneiro, M.O., et al. 2002. From FastQ data to high-confidence variant calls: the Genome Analysis Toolkit best practices pipeline. *Current Protocols in Bioinformatics*, 11:11.10:11.10.1–11.10.33.

Quinlan, A. R. and Hall, I. M. 2010. BEDTools: a flexible suite of utilities for comparing genomic features. *Bioinformatics* 26, 841-842.

Untergasser, A., Cutcutache, I., Koressaar, T., Ye, J., Faircloth, B. C., Remm, M., Rozen, S. G. 2012. Primer3—new capabilities and interfaces. *Nucleic Acids Research* 40, e115.

Appendix SA: PRIMER3 v2.3.6 configuration file containing the parameters used for primer design in this study.

Primer3 File - http://primer3.sourceforge.net
P3_FILE_TYPE=settings

P3_FILE_ID=Default settings of primer3 version 1.1.4
PRIMER_TASK=pick_detection_primers
PRIMER_PICK_LEFT_PRIMER=1
PRIMER_PICK_INTERNAL_OLIGO=0
PRIMER_PICK_RIGHT_PRIMER=1
PRIMER_NUM_RETURN=1
PRIMER_MIN_5_PRIME_OVERLAP_OF_JUNCTION=5
PRIMER_PRODUCT_SIZE_RANGE=80-200
PRIMER_PRODUCT_OPT_SIZE=0
PRIMER_PAIR_WT_PRODUCT_SIZE_LT=0.0
PRIMER_PAIR_WT_PRODUCT_SIZE_GT=0.0
PRIMER_MIN_SIZE=18
PRIMER_INTERNAL_MIN_SIZE=18
PRIMER_OPT_SIZE=20
PRIMER_INTERNAL_OPT_SIZE=20
PRIMER_MAX_SIZE=24
PRIMER_INTERNAL_MAX_SIZE=27
PRIMER_WT_SIZE_LT=1.0
PRIMER_INTERNAL_WT_SIZE_LT=1.0
PRIMER_WT_SIZE_GT=1.0
PRIMER_INTERNAL_WT_SIZE_GT=1.0
PRIMER_MIN_GC=45.0
PRIMER_INTERNAL_MIN_GC=20.0
PRIMER_OPT_GC_PERCENT=50.0
PRIMER_MAX_GC=55.0
PRIMER_INTERNAL_MAX_GC=80.0
PRIMER_WT_GC_PERCENT_LT=0.0
PRIMER_INTERNAL_WT_GC_PERCENT_LT=0.0
PRIMER_WT_GC_PERCENT_GT=0.0
PRIMER_INTERNAL_WT_GC_PERCENT_GT=0.0
PRIMER_GC_CLAMP=2
PRIMER_MAX_END_GC=3
PRIMER_MIN_TM=55.0
PRIMER_INTERNAL_MIN_TM=57.0
PRIMER_OPT_TM=60.0
PRIMER_INTERNAL_OPT_TM=60.0
PRIMER_MAX_TM=60.0
PRIMER_INTERNAL_MAX_TM=63.0
PRIMER_PAIR_MAX_DIFF_TM=3.0
PRIMER_WT_TM_LT=1.0
PRIMER_INTERNAL_WT_TM_LT=1.0
PRIMER_WT_TM_GT=1.0

PRIMER_INTERNAL_WT_TM_GT=1.0
PRIMER_PAIR_WT_DIFF_TM=0.0
PRIMER_PRODUCT_MIN_TM=-1000000.0
PRIMER_PRODUCT_OPT_TM=0.0
PRIMER_PRODUCT_MAX_TM=1000000.0
PRIMER_PAIR_WT_PRODUCT_TM_LT=0.0
PRIMER_PAIR_WT_PRODUCT_TM_GT=0.0
PRIMER_TM_FORMULA=0
PRIMER_SALT_MONOVALENT=50.0
PRIMER_INTERNAL_SALT_MONOVALENT=50.0
PRIMER_SALT_DIVALENT=0.0
PRIMER_INTERNAL_SALT_DIVALENT=0.0
PRIMER_DNTP_CONC=0.0
PRIMER_INTERNAL_DNTP_CONC=0.0
PRIMER_SALT_CORRECTIONS=0
PRIMER_DNA_CONC=50.0
PRIMER_INTERNAL_DNA_CONC=50.0
PRIMER_MAX_SELF_ANY=5.00
PRIMER_INTERNAL_MAX_SELF_ANY=12.00
PRIMER_PAIR_MAX_COMPL_ANY=5.00
PRIMER_WT_SELF_ANY=0.0
PRIMER_INTERNAL_WT_SELF_ANY=0.0
PRIMER_PAIR_WT_COMPL_ANY=0.0
PRIMER_MAX_SELF_END=3.00
PRIMER_INTERNAL_MAX_SELF_END=12.00
PRIMER_PAIR_MAX_COMPL_END=3.00
PRIMER_WT_SELF_END=0.0
PRIMER_INTERNAL_WT_SELF_END=0.0
PRIMER_PAIR_WT_COMPL_END=0.0
PRIMER_MAX_END_STABILITY=100.0
PRIMER_WT_END_STABILITY=0.0
PRIMER_MAX_NS_ACCEPTED=0
PRIMER_INTERNAL_MAX_NS_ACCEPTED=0
PRIMER_WT_NUM_NS=0.0
PRIMER_INTERNAL_WT_NUM_NS=0.0
PRIMER_MAX_POLY_X=4
PRIMER_INTERNAL_MAX_POLY_X=5
PRIMER_MIN_THREE_PRIME_DISTANCE=-1
PRIMER_PICK_ANYWAY=0
PRIMER_LOWERCASE_MASKING=0
PRIMER_EXPLAIN_FLAG=0
PRIMER_LIBERAL_BASE=0
PRIMER_FIRST_BASE_INDEX=0
PRIMER_MAX_TEMPLATE_MISPRIMING=-1.00
PRIMER_PAIR_MAX_TEMPLATE_MISPRIMING=-1.00
PRIMER_WT_TEMPLATE_MISPRIMING=0.0
PRIMER_PAIR_WT_TEMPLATE_MISPRIMING=0.0
PRIMER_LIB_AMBIGUITY_CODES_CONSENSUS=1
PRIMER_MAX_LIBRARY_MISPRIMING=12.00
PRIMER_INTERNAL_MAX_LIBRARY_MISHYB=12.00

PRIMER_PAIR_MAX_LIBRARY_MISPRIMING=24.00
PRIMER_WT_LIBRARY_MISPRIMING=0.0
PRIMER_INTERNAL_WT_LIBRARY_MISHYB=0.0
PRIMER_PAIR_WT_LIBRARY_MISPRIMING=0.0
PRIMER_MIN_QUALITY=0
PRIMER_INTERNAL_MIN_QUALITY=0
PRIMER_MIN_END_QUALITY=0
PRIMER_QUALITY_RANGE_MIN=0
PRIMER_QUALITY_RANGE_MAX=100
PRIMER_WT_SEQ_QUAL=0.0
PRIMER_INTERNAL_WT_SEQ_QUAL=0.0
PRIMER_PAIR_WT_PR_PENALTY=1.0
PRIMER_PAIR_WT_IO_PENALTY=0.0
PRIMER_INSIDE_PENALTY=-1.0
PRIMER_OUTSIDE_PENALTY=0.0
PRIMER_WT_POS_PENALTY=1.0
PRIMER_SEQUENCING_LEAD=50
PRIMER_SEQUENCING_SPACING=500
PRIMER_SEQUENCING_INTERVAL=250
PRIMER_SEQUENCING_ACCURACY=20
PRIMER_WT_END_QUAL=0.0
PRIMER_INTERNAL_WT_END_QUAL=0.0
=
