# Supplementary material for: Diagnostic single nucleotide polymorphism markers to identify hybridization between dromedary and Bactrian camels
Source: Conserv Genet Resour. 2015 Jan 13;7(2):329–32. doi: 10.1007/s12686-015-0420-z (PMC4486411; doi:10.1007/s12686-015-0420-z)
Supplement: Supplementary file 2 — Supplementary material 2 (PDF 32 kb) [file 12686_2015_420_MOESM2_ESM.pdf]

Online Resource 2

"Diagnostic single nucleotide polymorphism markers to identify hybridization between dromedary and Bactrian camels
"

In Conservation Genetics Resources

"Emily Ruiz, Elmira Mohandesan, Robert R. Fitak and Pamela A. Burger*
"

"Institut für Populationsgenetik, Vetmeduni Vienna, Veterinärplatz 1, 1210 Vienna, Austria
"

*Corresponding author:  pamela.burger@vetmeduni.ac.at

Primer	Scaffold	Position	Reference	Alternate	Fragment	Left Start	Left Length	Left TM	Left GC%	Left Sequence	Right Start	Right Length	Right TM	Right GC%	Right Sequence	Product Length
HP405	NW_006211106.1	5030405	G	C
GTACAGTGCTTGGCACATATAAGAGTGCCATTAGTGGTAGATTCATTCATTCATAGCCAATTCATCCA
TCCACCCATCCATTTATTCATTTATCAAAACAAACATTTATTGAAGGCCTCCTCTATGCCAGGAGCTT
TTCGAGTAGCTGTTGATAAGATAGACAAAGGTTTTGCCCACGTGGATCTCCTATTCTTCTCAAGGGAG
GTAGACAGTAAAGAGGGAAACAAAACTGCAGTGAGTTCTCTGTGCTGGGAAGCAAAAAATCACGGGGC
CGGATTGGGGAGCCGTGGAGGTGGAAGTGGGGCTTGCTCTTCAGCGGTTGCTCGGAGTGGGTAGAGGA
GTCCTCTCCGAGGACTTGACATTTGAGTGGAAACCAGAGCAATGCGAAGTCAGGCATGTAC
126	20	59.22	55	CCAGGAGCTTTTCGAGTAGC	250
20	57.22	55	CAGCACAGAGAACTCACTGC	125
HP900	NW_006211075.1	294900	G	C
CATCACGTGGGAACGTGTGTAGAGGCTTTGTGCATGGAGGTACTGGACACTGATGATCTCTGCTCCAG
GAGAGGGATGCTGAACTGCAACACGGAAATAGTCTTGTCTAGAGAAAAAAATACCACATGCTCAGGTA
TCTGGCACTCAGAGCCCAGGTGCCAGCTTTCTAAGATCTGAGAAGGCTAGCTGCTAGCATCCAGGATC
TTTGCAGGAAATTTGGATTTGGGCTGTAGCACAAGGAATCCCACTCAGTGAAAAGCTGAATTTGAGCA
CCAACCCTTCTGTTTACAGCCAGACAACTTCAGTTTTTGGAGTCATAAATCGGATGCTCCTGCTTATC
TCTTCACAATGACCTGGGAATTTCACCAATTTTTGCGTTAGTTAAAGGTGGATGTCTGTCT
121	20	59.11	55	CCACATGCTCAGGTATCTGG	245
20	59.7	55	GGGATTCCTTGTGCTACAGC	125
HP288	NW_006211252.1	214288	T	A
ACTCTCTTTCAGGCATTGTGCAAAGCATTTACCCACATTTTCTCATTAGGTGGTTGTGATGATTAATT
TTATGTAACAACTTGGCCACAGGGTACTTTTGGTCAAACATAATTCTGGGTGTGTCTATGAGGGCGTT
TCTGCACGAGATCAGCATTTGAATTGGTGGACTGAGTAAAGCACTTTGTCTTCCCTACTGTCGGTGAA
TCTCATCGAATCACTGAATACTCGAACAGAACAAGAAGGCTGAGTAAGAGGGAATTTCTCTTGCTTGA
CTACTTTGAGCTGGGACATCAGTCTTTTGTGGCTTTGAGACTCAATGAAACACTGGTTCTTTTTGGGT
CTCAAGTCTGCCCACTTTTGGACTGGAGCTACACATTTATTCTCCTGGGTCTCCAGCTTGC
121	20	59.84	55	GTCTATGAGGGCGTTTCTGC	245
20	58.65	50	CAGCCTTCTTGTTCTGTTCG	125
HP597	NW_006211126.1	6065597	A	C

AAACTGGAGTAACCAAAAAATCCCTGAACTGGCCAGTGCATCCAGAGGCCTAGTTCACCTGCCCCCCG
CCCAGCACAGGCTGGTGCACAACAGTTGCCCCGTGACTTTAGCTGAAAATGAACAGTTTGGGTTTGGG
GTGGGGAGAACTAACCTATGAATACACAGACAGACCTAAGAAACAGATTTTAAAATAGGCCCAAATGC
ATATGGAAATTCAGCGCCCTATAAAGGTGACATCGCGGATGAAGGGGGAAAGACCTTTCAGTGAGTGG
TGCTAGAGTCACTGGAAGAATGAAACCATCTAAGAACTAGAATGAAATGAAAAATGAAGGAATTCCTG
TAAACCTGAAAGTCAGAAAAACTTTCCTGATTTCCGAATCCAGGTCTATTAAGGGAAAAAA
116 20 59.69 45 ATGAACAGTTTGGGTTTGGG 240
20 58.27 50 CGCGATGTCACCTTTATAGG 125
HP264 NW_006211022.1 519264 T C
ATGTGGAAGATAAAAAAAAAGCACCTAATCACAACAACAAAGTGTCTCCTCTTCAGAAAGCCTGGGGA
GTGGAATTATGTACACTTTTTCTATAGAAATATGGATAGCATCCTCATATTTGTTATGGACAGAAACT
TTGTGTCTCCCCTAAACTCATATGTTGAAATTTTAACCCCCAATGTAGCATTTGGAAGGTGATTTGGT
CATGAGGGTGGAGCCCTCGTGAATAGGATTCATGCCCTTACCAAAGGGACCCCAGCGAGTTCTCCTAT
TGCTTCCACCAGGAAGCAGGCTCTCAGCAGACACTGAGTGTGCTTACACCTTGACCTTGGACTTTCCA
AGCCTGCAGAATCGTGAGAAATACATTTCTTTTGTTTATGCCACCTGGTCTGTAGTATTCT
124 22 59.23 45.45 TGGACAGAAACTTTGTGTCTCC 248
20 59.76 45 TTTGGTAAGGGCATGAATCC 125
HP206 NW_006210212.1 501206 C G
CTCAGTGAATTAGGAGGCAAGATCAGAAGTGGAAAGGGGATGTGGGGTACAGGTTGGAGTTTGAGTAG
AAGAAAGTGTGAAATAATTGTTTAGGAGAGTGGAATAGTAAATGGACTAGAGATGTTGTCAGACTGTT
AGGCATTGCTAAGAGCCCGCCGAAATTGGTCAGAGATTTAAAGTGAGACTAGTCATCATACGTTCGTG
ATTTTCTCTGGGATCATTCAGTCGGGTTAGATGGAGACTTGGATGTAACCTAGGGTTTTGCCAGGCAA
AAAGGACAGAGGGAGACTACACGTAAGGGTGTGACGATGGTCTGTAGGGTGTAAGCCGAGTAGGATGA
AGGAAAGGACGTGAGGAGGGGAAGGGAATAGGGGCAGGTGGTAGAATCACCATGTTGGGGA
124 21 58.94 47.62 TGTCAGACTGTTAGGCATTGC 248
22 59.83 50 CATCCAAGTCTCCATCTAACCC 125
HP429 NW_006210666.1 2288429 A C
ATTCAGACCTCAGCTAGAGTCACTTTTCTAGATGGACTTTGTCCATCTGGAGAATGGGATCTCATGGA
CAGACAGCCTGGCCAGGATCCAGGAAGGCACCAGTGTGGTGACAGGCATCACCAGAGCTGGGCATGGC
AGCCTCAGTTGGGAGATGGGCAGGCATACAAACTAACCCAATATGGGAGTGGGGATGGGCAGTGACAA
AGTTGGTGGCACAGGGTGTGCTGGGAGCATGCATGGGGAAGGGGGACTCACCCCAGCCTGAGCCAGAA
AGAAAAGCTTCTTGGGGAATGCAATGCCTGGGTTGATGCTTCAAGGATGAATAGGCACTTGGGCTGGA
GTAGAGATTTCAGGGATGGTAGGCTTTAGGGAGACTGGGAGGTCTTCTCTTCAGAGTCTGC
155 20 57.24 50 GCAGGCATACAAACTAACCC 279
20 59.19 50 GCTTTTCTTTCTGGCTCAGG 125
HP633 NW_006210489.1 4279633 G C
GTTTCCTACTACCGTTAAAGTCTGATGTGTCTTCAACAGCTCTCCCTGGTGACTCTCCGAAGCAGTCC
TGCAGGGATCTGGGATCTTCCGCTATCTGGCTGTGCCATTTCGGAATCGAAAAAGTAAGCATGTAGAA
GGTTTGCATAGGGGATGTCTGATGGCCAGGTCTGGTGATGGCTTACCTCACTTCTGCTTTTATTGCAT
CGGCCAAAACCTAGTCACACGGGCCCACCCAGATGCAAGAAAGGCTGGGAGATGTCATCATGTGTGCT
TGGGAGGCAAATGAAAGTCTGATGACCTTGGAGCATGGTTTCCACCAAAATTATCATTTCCTCAGGTA
AGCCTTTTGTGACTTCCTGGTGGGATCTAACAGGTTAAGGCGCCCATTTCCTCTGTTTTTC
126 22 58.79 45.45 GCATGTAGAAGGTTTGCATAGG 250
19 59.53 52.63 CAGCCTTTCTTGCATCTGG 125
HP930 NW_006210745.1 3459930 C A
ACAATCTAGAAGAAATGGACACATTTCTAGAAACATAAAGCCTGCTGAGATCAAATAAGAAAAAAACA
GACAATTTGAACAGACTGACCACTAGCAGTAAAATTAAATTTGTAATTTAAAAAACTCCCAGGAAACA
AAAGTCCAGAGCTAGTCAGCATCACAAGGGAATTCTACTAAATATAAAAAGAAGAAATACCTATCCTT
CTCAAACTATTCCAAAACATTGAAGCAGACAGAACACTCCCAAATTCATTCTTTGAGTCCACCATTAC
CCTGAAACCAAAACCAGATGAAGACACTACAAAAAAAAAAAAAAAAAAAAAAAAAAAAGAGAGAGAGAG
AGAGAATTACAGGCCAGTATCTTTGATGAATATAAATCCTCAACAAAATGTTAGCAAACTG
123 20 58.62 50 CTCCCAGGAAACAAAAGTCC 247
20 58.85 50 TTTGGGAGTGTTCTGTCTGC 125
HP458 NW_006210457.1 11458 T C

AGTATTTGTTCTCAGTACTCAGTTCCTGACATAAACACTTCTAAGACTCTGGGATCTCCAGAGTGATA
AGAATCTTTCTGAATGCTAATGAGGTGACTGGTGTGTGTGGGGGGGCCCAGGGAGCATCAGGATGGGG
TCCTGTGACCAGACAGACCAAGGCATGACTGGAGGGTTGGAACTTTCGTCCACCCCATCCTGACTGTC
AGAAAGGGGATGGGGGCTGGAGACTGAGTTAATCACCAATGACAAATAAAATATCCATAAAGACCCTA
AGCCACACGGTTCAGAGCACTTCCACTTTGGCACACACATCAAGGTTCTAGGAGGGTGAAGTGTCTGC
AGAGGACACAGAAGCGCCACTCACCCCTTCACCCAAGCTGTGCCTTCTCCATCCTTCCATC
139 20 59.7 55 TGTGACCAGACAGACCAAGG 278
21 59.1 47.62 TGTGGCTTAGGGTCTTTATGG 140
HP379 NW_006210464.1 1151379 G A
AAAAACCTGTCTCAGTCAGTTCAAGCTGCTTTAACAAAGTATCTTAGACAAGCGACTTAAAAACAACA
GAAATTTATTTCTCACAGTTCAGGAAGCTTGAAATTCAAGATCAGGATGCCATCATGTCAGGTTCTAG
TGAACACCCTCTTTCAGTTGTTAATTACCAATTTTTAGTTGTATCGTCACATGGAAGAAAGAGAGTGA
GCTAAATCTTCAGCATCATTTTATAAGGGCACTAATCCTATTCATGAGAGCTCCCTCCTCATAAACTA
ATTACTTCTCAAAGGTCTCACCTCCATATACCACTGCTTTGGGGGTTTCATTGTATGAACTTGGGGGG
ACACAAACATTCAGTCCATAACAACACCATAAGGTCAGTATCCTTGTGGAGACAGAACCAT
111 19 59.44 52.63 AGGATGCCATCATGTCAGG 260
21 57.96 52.38 GAGGGAGCTCTCATGAATAGG 150
HP501 NW_006211169.1 218501 A T
AAGCAGGCAATCAGAAAGGGCCTAGCCTGTCACATGAAAGAATGTGGACTATGTTATGAGGAAACTGG
AGAGTCACTGAAGGGTTTTAAGCAGAGAAGTGACATTTTGAATTTTATAAAGATCTGTCAGAGTATGA
TATAGAGAATAGATTGGGGAGCAAGCAAAACAGACTAGAGAGAATCTTAAGGAGAATATTGCAAAAAT
GCATAGAAATGAAAAATAATGGTGTTTTGCACCACTATTTGTAGCCATAGGGATGGAGAAGAGCATAT
GTACTCAAGAGAGACTCCAGTATCAGAATGGGACAAGCCGTGGTGAATGACTGAGGCTGGCTGAAGGA
GAGAAAGGAATTGAGAATGAATCTCAGTTTTTGCCTTGACGGGCCTTTCTGGATTAGGCAG
142 20 58.74 50 GAATAGATTGGGGAGCAAGC 266
20 58.69 55 CTCTTCTCCATCCCTATGGC 125

HP651 NW_006210190.1 16651 T G
TATCTTCTCATACTCCATGGTTTGCTTTTTCAGTCTTAAGAGTGGCTTTTGAGAAACAGAAAACCTTT
ACCATAGTATAGTCCTTTTAGTAATCACCAACATTTTCCCTGATACTTATTGTGCTTTTTATGACTGC
TTAGTGAGAGCAATCCCAGAATACTTTAATTTTATTCATGACTGAGAGGATTTGGAGCTGGCTGTTCT
TTTTCTGATTTACTAATCTCATTAGTGCATAGCCCTTCTGAGTCTCCCAACTCTAAGCCTGGGGTGGA
CGTGGTGTGATTATCAGAATCTCTTTTCTTTGAGGGGCTTTGGTCTCCATTTTTTTTCTCCTAGCCAT
ATGAGCCTGGCAGAAGCTCTGCTCAGTTTCTTACACTCTCAAAATGCCTTGACTATAATTG
132 21 59.09 52.38 CTGCTTAGTGAGAGCAATCCC 255
20 58.31 55.00 AGTTGGGAGACTCAGAAGGG 124
HP168 NW_006210425.1 299168 C T
GGAAAATTCATAGCCTGAAATATTCATGTTAATAAACAAGATGCACTGAAAATAATTGAATTAAATAC
TCCTACACTTCGGATACCAGTTCCAGTGTATGTGTTTTTGTAACAGCCCCAGACAGTTCTCCAGCACC
AGCTGGGGGTCTGCAGTTCAACCAGATTCCGTCCCACCCCCCTGGAGACGTCAGGTTAAGGGCTCGGC
CCCACAACGTGGCCCCGTCATCTGGGCTTCTGCCCAGCAGCTGTAGACTGGGGGTTCCCATAACCGCC
TCCTCTGGTTTGACTGACTTGCTAGCCTGGCTCACAGAACCCAGAGAAGCGTTTTCCCTACGAGATTA
CGGATTTATTGTAAAAGGCGGTAACTCAGGAAGCAACACAGACAGCGAGGCCTGGGTTCCT
146 20 58.29 50 CTGCAGTTCAACCAGATTCC 293
21 59.9 52.38 GCAAGTCAGTCAAACCAGAGG 148
HP906 NW_006210403.1 854906 C T
TGTCCTGTGGCAGCTGTAACAAGGTACCCCAAACTGGGCGACTTACAGCAACACCCGTAGTGGCTGAA
CTATGTCCCCTCAAAATTCGTATGTTGAAGCCCTAACTCGCAGGATCTCAGAATGTGACTGTGTTTGG
AGATGGGGCCTTTAAAGAGGTAATTAAGGTTAAATGAGGTCCTTAGGGTGGGCCTGAATCCATTCGGA
GAGGGGTCATTCTAGAAAGAGGAGATTAGGGCACAGACACAGAAGGACAGCCACGTGAGGACACTGGG
AACAGGCCGCCTTTGGCAAGCCACAGAAAGAGGCCTCAGGAGATGCCAACCCTGCTGACCCCTTGTTC
TTGGACTTCCAGCCTCCAGAGCTGTGAGTTAATAAATTCCCGTTATTCAAGCCACCCTGAT
136 20 59.55 50 AGATGGGGCCTTTAAAGAGG 243

20 57.16 55 GTGTCTGTGCCCTAATCTCC 108
HP420 NW_006210558.1 282420 C T
TTTTTCCTATTTTATGGGATTGAGTAGTGTCCACCCAGAACCTCAGGATGACCTATTTGGAAATAGGG
TCATGGTAGATGTAATTAGTTACATTAAGATGAGGTCATAATGGATTGGGGTGGGCACTAAATCCAGT
GACTGGTGTTTGTAAGAAGGCCATGTGAAGAGAGACAGACAAAAGAGTTGAGCTTTCACAGGCCAAG
GAGTGCCAGAGATTGCCAGCAACCTCCAGAAGCAAGGAAAGGGGCAAGGAAGGATTCTTTGCTTAATT
CTTCAGAGGGACTGTGCCCCTGCTGAAACCTTGATTTTAGAATTCTATCCTCTAGAACTGGGAGAGAA
TAGATGTCTGTTGTTTTCAGCCACTTGGTTTGTGGTAATCTGTTTTGGTGGCCCTAGGAAG
121 21 59.61 52.38 GGCACTAAATCCAGTGACTGG 264
19 57.32 47.37 CAAAGAATCCTTCCTTGCC 144
HP098 NW_006210403.1 496098 G T
AGACAGCACCCTCTTGTCAGGACATTGAAGTGTGCAACGATTTCTTAAAGAGAACGCCAAAAGGGCTT
ACCCTGAAAGAAGACACTCGAACATTGGGCTTCATTAAAGCTGAAAACTTCTTTTCAGGAGCAAGCAC
CATTAAGGACGAGAAGAGACAACTCATAGACGTGGATGAGATGCCCTCATTGTCTCTGACAGAGGAGT
CGTGTAAATACAAGTAAAAAGAGAAACATAGTCCAATTAAAAGATGGACAACAAGAAAAGGGAACCCT
CCTACACTGCTGGTGGGGATGTAAATTGGTGCAGCTACTATGGAGCACAGTCTGGAGGTTCCTTTAAA
AACTGAAAGTAGGGTTACCATACGATCCAGCAATCCCACTCCTGGGCATATATCTGGAAAA
124 20 59.34 50 AGGAGCAAGCACCATTAAGG 269
21 59.83 47.62 GGTTCCCTTTTCTTGTTGTCC 146
HP148 NW_006210453.1 347148 A C
AGATTTATATCAGAGAATGTTTTGCCTATGTTCTCTTCTAGGTTTATGGTGTCCTTATGTTTAAGTCT
TTAAGCCATTTCGAGTTTATTATTGTATATGGTGTGAGGGGGTGTTATAAATTCATTAATTTACATGC
ACCTGTCTAGCTTTCCCAGCACCACCTGTTGAAGAGACTGCCTTTTCTACATTGTGTATTCTTGACTC
CTTTGTCAAAGATTAATTGACCATAGATGTGTGGATTCATTTCTAGACTCTCTATTGTGATCCACTGA
TCCACATGTCTGCATCTGGGCCAATTCCATGCTGTTTTAATTATTGTAGCTCTGGAGTATTGTCTGAA
GTCTGGAAGGGTTATTCTTCCAGCTTCATTTTCTTCAGTATTGCTTTGGCATTTCTGGGTC
157 20 58.44 55 CCACCTGTTGAAGAGACTGC 283
19 55.6 52.63 CAGACATGTGGATCAGTGG 127
HP710 NW_006210331.1 1907710 G C
GTAATTTGCATAGATATAATTCAAGTAAATTTCTCTGAACACAAGTACATACTTTCTCAGAGTGAAAA
AAGTTTACACAAAATAACAGACAATGAATTTTAAAAGATCTACCCATACCAAGGCACAATCCAGTAAC
ATAAACAACAAACAAATTTTAAAATAAAAATATTAAAAGCTTCTTGGGGGCAAGGGATGGGAAAGACT
ATAATTTTCGGCTTATAATCCTAATATCCCAACAATCCATCAATCACATACAAAGGTTAAAAACAAAA
AAAAGACATTTTCATACAAGCAAGCTCTAAAAAATTCTACTGTCATGCATTCTTAAGAAATTACAAAG
AACGTGCCTCATCAAAATTAATACTGAGGCCCCACTATCATCCTCCATCCCTGTTCTGCTT
111 19 58.78 52.63 CCATACCAAGGCACAATCC 250
20 58.62 45 GTGATTGATGGATTGTTGGG 140
HP638 NW_006210469.1 1696638 A G
TTGTAACATGCTCCAAGAAGTAAGGGCATATGCTCTTGAAACAAATGGAAAGACAAGAAGTCTCAAGA
GAGAAATAGAAGTTCCTGAAAAAAGTACAAAATGGAAATTTTAGACAGAAAAAAATACAGTAGCAGAA
ATTAAAAGAATCTACAAGATGGGCTTGATAGAAGAATGGAGAAGACAGAGGAGCTGACTGGAAGACAG
GTCAGTAGGAAGTACACTTCATAAACAACACAGAAAAGTGTTGAAAAATTAACAGAGCCTCAGATACC
TGTGGAACAAGATCCAAGTTCCACCATTCGTGTCAGAAGAGTCTTAAATAGAGAGGACAGAAAGTGTC
CTGTGGGAAAATATTTGATGAAATAATGGCAAAAACTTCCAAATTTTGGTGAAAGACATTA
153 22 59.93 45.45 GATGGGCTTGATAGAAGAATGG 303
19 59.94 52.63 CACGAATGGTGGAACTTGG 151
HP294 NW_006210344.1 1536294 C A
ATCTCTATTTCCTCATAGGATATATGCGGGGGTAAATAAAGTTACATGTAAGGCATTTAGCACATAAT
AGGCATTTAATTCATATTAAGTTTCATAGCACCCCAAATTTTTCTTTTGTAGCACTAGCCACAGTTGC
AGCTAAATAATTTTGGAATTATTTATTTTATGTTTTTCTTCTCGTAAGCTTTGTGAGGCGAGGGCCCA
AGTTTATCTCTGGCTTTATTCCCAGCATCCAGCACAGCCCTTGGCATGGCATATGAGATGCTCAATAA
ATACTTGTTGAATGAATGAAAGTGACCCACCTCCTCTTTTTAACGCATCTTCCTTGCCTTGAAGGACA
GTGTTTTGAACTTATTTCCTTTGGCCCATTTGAGAATTAATCCACATGTTACTTGAACTTT
115 21 59.16 52.38 TGTAGCACTAGCCACAGTTGC 259

18 58.97 50 TCATATGCCATGCCAAGG 145
HP628 NW_006210373.1 1346288 C A
CTGGTGGTTACCAGCGGCCCCTGTGTTCCCGGGCTGTGGCTGCATCAGCCCAGGCCCTGCCTCTGCCT
TGGTGGCCTCCTCTGTCTCCACGGCTTCTGCTCTCTGCTGGCGCCAATGTCCCTCTTCTCACAGGACA
CAGTCACGTGGGATAACGGCCCATCCTCTAGCAGCACGACCGCCTCTCACCTTGATCACAGCTACGAA
GACCGTATTTCCAAACCCGGTCCCGCTCACAGGTACCGAGTGTTGGGACGTGGACATGCCTTTGGGGG
TGGACACAATTCAACCCCAGACAGTGATGAGCTGGGGCAGGACAAGGTGCCATGAGGGACTTAGAGGA
ACCCAGGCCCTCACACAGGCGCCCTGGTGAACAGTGGCCCAGAGTGTGTAAGTCAGACGTG
123 21 59.92 52.38 TTCTCACAGGACACAGTCACG 247
20 57.80 55.00 AACACTCGGTACCTGTGAGC 125
HP767 NW_006210904.1 1161767 G T
CTCTCCCTCTAAAAGGTAAAAAATTAAAGATAAATAAAATTGGTGATACAGAAGTTTTCTTAATAGAT
AACTGACTCTATTATCTAATTACCACCCAAAAGTAAAGCTTTCAATTCTAATAATAATTCGAAGTTAT
AATCTTTTGCTAGACCCTCCACCTGACTTTAAACAGCAGCATAGCTAACATCTTCATACAGCAGGAGA
ATTCATTTAGTGATTTACATGTATGTTTTACAGCTCAACTAATTTTTCTTGGAAGCCATTGCAGGAAG
TTACGTTACTCAAAAAGTAGATACTGTTTGAGTTTTTCAATGAGATTAAGGGAAAAAAAAAAAAAACT
AGTTGGCATATCTGTTTGGTTAGACGTCTTTTGAGTATTTTCCAGACTTATTTTTAACCGT
152 21 56.63 47.62 CTCCACCTGACTTTAAACAGC 276
19 59.29 52.63 CGTAACTTCCTGCAATGGC 125
HP820 NW_006210999.1 173820 C G
TAATCTACTGTGTCTTTAGTTCAGTTTATTGAGTGTTTATTTAGGTTTCATTTTCAGAGCTTAAAGTA
CTTTGTTTATTTGGGAGAAAATCCCAGGAAGGACCACTACTAGATGAGGAAATGAGAGAGGGGAGAGA
AGAGCATGTACTACTGAGCTGATAACTGCTGTGGATAACCAGGGCTCAATCCAGATGGAGAACTCTGG
GTGGCACTGTAAGAACAATGCCAATCAGGGTGAGGAGGGTATAGCTCAGTGGTAGAGTGCCTGCTTAG
CATGCACAAGGTCCTGGGTTCAATCCCTAGTATCTCCAAACAAATAAATAAACCTAATAACCTCCCTC
TGCCAAAAAGAAAGAAAAAAATAAAGTTAAAAATGTCTTTAAAATAACTTAAAAAAAAAAA
149 21 58.69 52.38 CTGAGCTGATAACTGCTGTGG 273
20 58.28 55.00 TGCTAAGCAGGCACTCTACC 125
HP662 NW_006211260.1 2760662 A T
ACAAAAGCAATTTTTAAAAACTGCAAAAACACGAACAGGTGGAGGCTAAACAATATGTTACTAAACAA
CCAATGGACCACTGAAGAAATCAATAAGGAAATGAAAAAATATCTAGAGACAAATAAAAACAAAAACA
AGAAGATCCAAAACCTATGGGACACAACAAAATCAGTTTTAAGAGGGAAGTTGATAGTGATACAAGCT
TACCTCAAGATATAAGACAAATCTCAAATAAACAACCTAACCTTACACCTAAAGCAGCTAGAGAAAGA
AGAACAAACAAAATCCAAAGTTAGCAGAAGAAAAGAAATCATAAAGATCAGAGCAGAAATAAATGAAA
TAGAGACTAAAATAAAATAGAAAAGATCAATGAAACTAAAAGCCGGTTCTTTGAAAACATA
137 20 56.07 45.00 GAAGATCCAAAACCTATGGG 261
23 58.60 47.83 GCTGCTTTAGGTGTAAGGTTAGG 125
HP324 NW_006211261.1 3675324 T G
TACTCACTCCCTCTGGTAGCCTCTGGGTATCCCTCCAAAACCATTGGAAGAGCTGTCTTTCCAAAGAT
TCTAATCAAGTTGCTCTTCTGCTTAAAACCCATTAGTTGCTTCCCATTGCTCATAGAAAAAAGTCCAG
ATTTTTACCGTTGCCTGAAAGGTGCTGTGTGATCTGGCATCCGCCTACCTCTTACACCATTCACTCTA
TGCCAGCCACACTGGTCTTCTTTTAATTCCTAGAATTCATCAGGAGACTTCCCAGCTCTTGGCCCTTA
CACATCTGTTTCCTCTGCCTAGAAGTTCATCTCATCTTCCCCCTCCCTTCCTGGAAAACTCCTTCTTG
TCCTCCAAGTCTCTATTGAAATGGCAGTTCATCAGGAAGAACTTCCTTTCCCCTCTTCCAG
153 20 58.72 50.00 AAAGGTGCTGTGTGATCTGG 277
20 59.70 55.00 GATGTGTAAGGGCCAAGAGC 125
