# Supplementary material for: Diagnostic single nucleotide polymorphism markers to identify hybridization between dromedary and Bactrian camels
Source: Conserv Genet Resour. 2015 Jan 13;7(2):329–32. doi: 10.1007/s12686-015-0420-z (PMC4486411; doi:10.1007/s12686-015-0420-z)
Supplement: Supplementary file 3 — Supplementary material 3 (PDF 61 kb) [file 12686_2015_420_MOESM3_ESM.pdf]

Figure S1: Example electropherograms (locus HP405) illustrating a diagnostic polymorphism between Bactrian and dromedary camels.

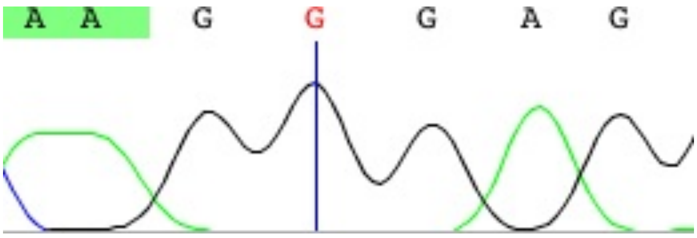

DC158 (*C. bactrianus*)

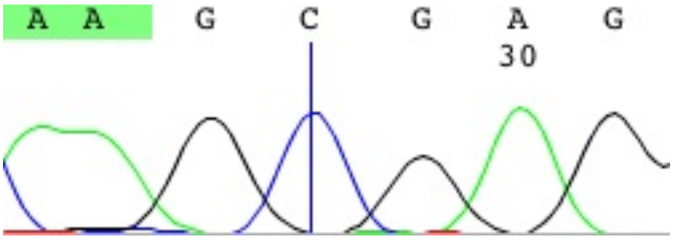

Drom814 (*C. dromedarius*)

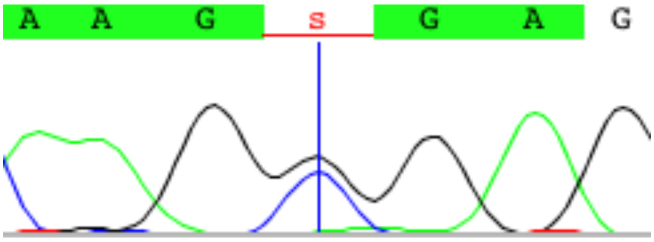

Hyb56 (F_1 hybrid)
